# Temperature‐mediated responses of carbon fluxes to precipitation variabilities in an alpine meadow ecosystem on the Tibetan Plateau

**DOI:** 10.1002/ece3.5439

**Published:** 2019-07-23

**Authors:** Ning Chen, Yangjian Zhang, Juntao Zhu, Jiaxing Zu, Ke Huang, Junxiang Li, Yaojie Liu, Nan Cong, Ze Tang, Li Wang, Yixuan Zhu

**Affiliations:** ^1^ Key Laboratory of Ecosystem Network Observation and Modeling, Institute of Geographic Sciences and Natural Resources Research Chinese Academy of Sciences Beijing China; ^2^ University of Chinese Academy of Sciences Beijing China; ^3^ CAS Center for Excellence in Tibetan Plateau Earth Sciences Beijing China; ^4^ College of Resources and Environment University of Chinese Academy of Sciences Beijing China; ^5^ Peking University Shenzhen Graduate School Shenzhen China

**Keywords:** alpine meadow, carbon fluxes, community composition, distinct response, precipitation, warming

## Abstract

Effects of climate warming and changing precipitation on ecosystem carbon fluxes have been intensively studied. However, how they co‐regulate carbon fluxes is still elusive for some understudied ecosystems. To fill the gap, we examined net ecosystem productivity (NEP), gross ecosystem productivity (GEP,) and ecosystem respiration (ER) responses to multilevel of temperature increments (control, warming 1, warming 2, warming 3, warming 4) in three contrasting hydrological growing seasons in a typical semiarid alpine meadow. We found that carbon fluxes responded to precipitation variations more strongly in low‐level warming treatments than in high‐level ones. The distinct responses were attributable to different soil water conditions and community composition under low‐level and high‐level warming during the three growing seasons. In addition, carbon fluxes were much more sensitive to decreased than to increased precipitation in low‐level warming treatments, but not in high‐level ones. At a regional scale, this negative asymmetry was further corroborated. This study reveals that future precipitation changes, particularly decreased precipitation would induce significant change in carbon fluxes, and the effect magnitude is regulated by climate warming size.

## 1 INTRODUCTION

While warming has been confirmed to be occurring as a part of climatic change, global hydrological cycle has been predicted to strengthen in the future (Stocker et al., 2013), especially for arid and semiarid regions (Ahlstrom et al., 2015). Studies reported that global semiarid ecosystems contributed 39% of the interannual variabilities of global terrestrial carbon sink (Ahlstrom et al., 2015). The alpine meadow ecosystem, as a semiarid ecosystem across the Tibetan Plateau (TP), experienced rapid climate warming and high variability of precipitation in the past decades (Xu, Wang, Zhao, & Yang, 2018; Zhang, Zhu, Li, & Chen, 2019). Changed precipitation may have even greater effects on carbon fluxes than warming in this region (Fu, Shen, & Zhang, 2018; Zhang et al., 2018). Therefore, understanding patterns of ecosystem structure and function in response to precipitation changes is critical for predicting their provisioning of ecosystem services (Nimmo, Mac Nally, Cunningham, Haslem, & Bennett, 2015).

Precipitation variability is a key driver of ecosystem structure and function for arid and semiarid ecosystems (Liu et al., 2012; Suttle, Thomsen, & Power, 2007). Ecosystems respond to precipitation regime changes through shifts in species composition, distribution, and abundance (Scott, Hamerlynck, Jenerette, Moran, & Barron‐Gafford, 2015), as well as water and carbon balances (Kulmatiski & Beard, 2013). For example, six percent expansion of vegetation cover bring about fourfold strengthened sensitivity of net carbon uptake to precipitation change in Australia (Poulter et al., 2014). Hence, in evaluating carbon fluxes responses to climate change, precipitation variations should be fully considered (Xia, Niu, & Wan, 2009), particularly alternated dry or wet seasons (Bonal et al., 2008).

Strong precipitation variability leads to asymmetrical responses of carbon fluxes to increased and decreased precipitation. Gross primary productivity (GPP) or net primary productivity (NPP) is reported to be much more sensitive to increased precipitation (Unger & Jongen, 2015; Wilcox et al., 2017; Wu, Dijkstra, Koch, Penuelas, & Hungate, 2011) or decreased precipitation (Luo et al., 2008; Zscheischler, Mahecha, et al., 2014; Zscheischler, Michalak, et al., 2014). Currently, much of our knowledge is centered around ecosystem structure and function in response to naturally occurring climatic variations (Zscheischler, Mahecha, et al., 2014), extreme precipitation experiments (Knapp et al., 2015), or synthesis analysis (Wilcox et al., 2017), while significant knowledge gap exists for some typical ecosystems. In a related synthesis analysis, 83 studies of experimental precipitation manipulations in grasslands were incorporated worldwide, but no single case on the TP (Wilcox et al., 2017). Based on the optimized model, a recent study had reported that TP ecosystem was more sensitive to drying than to wetting (Liu et al., 2018). In addition, plenty of studies evidenced influences of precipitation of both nongrowing season and growing season on ecosystem structure and function on the TP (Cong et al., 2017; Shen, Piao, Cong, Zhang, & Jassens, 2015; Shen, Tang, Chen, Zhu, & Zheng, 2011). Despite our growing awareness and concern, a vital knowledge gap exists about whether the sensitivity of carbon fluxes differs under precipitation increases versus decreases on the TP.

A growing body of evidences demonstrated that warming would export strong direct and indirect effects on carbon fluxes. Climate warming can alter plant community structure and composition (Botkin et al., 2007; Gedan & Bertness, 2009). The warming effects on carbon fluxes vary with plant species (Chen, Luo, Xia, Shi, et al., 2016; Chen, Luo, Xia, Wilcox, et al., 2016), functional groups (Niu, Sherry, Zhou, & Luo, 2013), and root depth (Zhu, Zhang, & Jiang, 2017). Warming also can indirectly regulate carbon fluxes through stimulating evapotranspiration, reducing soil moisture, and exacerbating water stress (Niu et al., 2008). Weakened soil water availability related to warming will exacerbate water limitations on arid and semiarid ecosystems, offsetting part of positive warming effects (Niu et al., 2008). This phenomenon is more likely to be associated with precipitation changes (Chen et al., 2019; Dermody, Weltzin, Engel, Allen, & Norby, 2007). Studies on the TP also revealed that the interactions between changes in temperature and precipitation would regulate ecosystem structure and function (Ganjurjav et al., 2018; Shen et al., 2014). Both direct and indirect effects of warming on carbon fluxes were related to their magnitudes, because low‐level warming and high‐level warming induce different changes in soil water availability (Chen et al., 2019), water use efficiency (Quan et al., 2018), and community composition (Li, Wang, Yang, Gao, & Liu, 2011). Therefore, warming could regulate the precipitation effects on carbon fluxes through modulating the water availability and community composition. To date, few studies have reported this phenomenon on the TP, and multilevel warming was even more uncommon.

Here, we conducted a field experiment to investigate effects of multilevel warming on carbon fluxes in an alpine meadow ecosystem across the TP. This study was conducted for three hydrologically contrasting growing seasons (dry in 2015, wet in 2016, and normal in 2017), which presents a unique opportunity to reveal how precipitation change affects carbon fluxes under multilevel temperature increasing scenario. Specifically, two main questions were set to be addressed: (a) How carbon fluxes respond to interannual variability of precipitation under multilevel temperature increases? and (b) Whether there exists an asymmetric response of carbon fluxes to increased and decreased precipitation under multilevel of temperature increases?

## 2 MATERIALS AND METHODS

### 2.1 Study site

The study area was conducted at the Tibet Alpine Grassland Ecosystem Research Station, which is operated by Institute of Geographic Sciences and Natural Resources Research, Chinese Academy of Sciences (31°38.513′N, 92°0.921′E, 4,585 m). The study area represents a typical alpine meadow ecosystem. The long‐term mean annual temperature and precipitation are −1.05°C and 434.3 mm (1955–2017), respectively. The growing season normally starts in mid‐May and lasts until mid‐September. The vegetation is dominated by *Kobresia pygmaea* (*K. pygmaea*), accompanied by *Potentilla saundersiana*, *Potentilla cuneata*, *Potentilla bifurca,* and *Stipa purpurea* (Zhu et al., 2017).

### 2.2 Experimental design

Open top chambers (OTCs) were used as passive warming devices based on the International Tundra Experiment design standard (Marion et al., 1997). The OTCs used in the current study were similar to those described in other studies (Chen, Luo, Xia, Shi, et al., 2016; Chen, Luo, Xia, Wilcox, et al., 2016; Dabros & Fyles, 2010). Warming effects were achieved through changing OTC heights, and they included control (C), warming 1 (W1), warming 2 (W2), warming 3 (W3), and warming 4 (W4) (*n* = 3 per treatment). The OTCs were set up in October 2013 and made of 6 mm thick solar transmitting material. They were conical in shape, with heights of 40 cm (W1), 60 cm (W2), 80 cm (W3), and 100 cm (W4). The top sides of each OTC were 80 cm. The bottom sides were 100 cm (W1), 110 cm (W2), 120 cm (W3), and 130 cm (W4) and covered a ground area of 2.60 m^2^ (W1), 3.14 m^2^ (W2), 3.74 m^2^ (W3), and 4.39 m^2^ (W4), respectively. The 15 plots were separated by a 3.5‐m buffer and arranged following a randomized block design.

### 2.3 Measurements of carbon fluxes

Ecosystem carbon fluxes were measured by an infrared gas analyzer (IRGA; LI‐6400, LiCor Inc.) attached to a transparent chamber (0.3 × 0.3 × 0.3 m^3^). When the conditions within the chamber achieved a steady state, 30 consecutive CO_2_ concentration recordings were obtained on each base at 2‐s intervals. In order to ensure the air uniformity in the static chamber, two small electric fans were installed inside the chamber and ran continuously to mix the air inside. During measurements, CO_2_ concentration was allowed to build up or draw down over time, from which flux rates were determined and net ecosystem productivity (NEP) was calculated. Positive and negative NEP indicated net carbon uptake and net carbon release, respectively. Following NEP measurement, the chamber was vented and replaced on each frame. The chamber was covered by an opaque cloth, and CO_2_ exchange measurements were repeated. Under the second set of measurements, light was eliminated (and hence photosynthesis) and the obtained values represent ecosystem respiration (ER). The sum between NEP and ER was treated as instantaneous gross ecosystem productivity (GEP). Ecosystem gas exchange was measured every 5–10 days at 9:00a.m.–12:00p.m. from May to September in 2015–2017. These measuring processes followed the same standards of a previous study in our experimental site (Zhu et al., 2017).

### 2.4 Measurements of community coverage

A 1 × 1 m frame with 100 equally distributed grids (0.1 × 0.1 m) was placed above the vegetation canopy to measure vegetation coverage (1 × 1 m). Grids with plants appearing over 1/2 of the grid were marked as 1, otherwise marked as 0. The coverage was mainly measured in middle and late growing season (Chen et al., 2019).

### 2.5 Soil temperature and water content

Soil temperature and moisture at 5 cm belowground were measured in the centre of the plots using Campbell CS655 sensors (Campbell Scientific, Logan, UT). Measurements of soil temperature and moisture were taken with 30‐min intervals, and averages of the forty‐eight measurements were stored as the daily averages. In each warming treatment (three plots), we installed soil sensors in two of them and took their average (Chen et al., 2019).

### 2.6 Regional precipitation and primary productivity products

The annual precipitation data with a spatial resolution of 1 km × 1 km from 2000 to 2015 were provided by the Cold and Arid Regions Science Data Center (http://westdc.westgis.ac.cn). The NPP data with a spatial resolution of 1 km × 1 km were calculated by the Carnegie‐Ames‐Stanford approach (CASA) from 2000 to 2015.

### 2.7 Quantifying sensitivity to precipitation

Sensitivity was calculated as the response range relative to the amount of precipitation variability (Knapp, Ciais, & Smith, 2017; Wilcox et al., 2017). The advantage of this method is that ecosystem responses are comparable after they are standardized by the range of precipitation variability:

(1) $${\text{Sens}}_{\text{dry}}=\left|\frac{X_{\text{dry}}-X_{t}}{{\text{PPT}}_{\text{dry}}-{\text{PPT}}_{t}}\right|$$

(2) $${\text{Sens}}_{\text{wet}}=\left|\frac{X_{\text{wet}}-X_{t}}{{\text{PPT}}_{\text{wet}}-{\text{PPT}}_{t}}\right|$$

where, $X_{\text{dry}}$ and $X_{\text{wet}}$ represent the productivity in dry and wet years, respectively. $X_{t}$ represents the productivity means across 2015–2017 and 2000–2015 for in situ measurement and remote sensing products at the regional scale. ${\text{PPT}}_{\text{dry}}$ and ${\text{PPT}}_{\text{wet}}$ represent the precipitation amounts in dry and wet years, respectively. ${\text{PPT}}_{t}$ is the precipitation means across 1955–2017 and 2000–2015 for in situ measurements and remote sensing products at the regional scale. In this study, the absolute value for the calculated dry minus mean and wet minus mean is required to be roughly equal (the error being <10%). The ${\text{Sens}}_{\text{wet}}>{\text{Sens}}_{\text{dry}}$ indicates positive asymmetry, with the opposite indicating negative asymmetry.

### 2.8 Statistical analysis

The one‐way ANOVA was applied to compare the sensitivity of carbon fluxes to increasing and decreasing precipitation under low‐level and high‐level warming. The normality and homogeneity of variances required by the one‐way ANOVA was examined by Shapiro–Wilk and Levene statistic, respectively. If the test failed, we employed the nonparametric tests. Repeated‐measures ANOVA was used to investigate effects of time‐of‐season on carbon fluxes over the growing seasons in 2015–2017. If the data met the assumption of Mauchly's test of sphericity, we used sphericity assumed to analyze the within‐subjects effects, otherwise Greenhouse–Geisser. The above statistical analysis was conducted with SPSS 22.0 (SPSS Inc.). In order to reveal the similarities among carbon fluxes responses to precipitation variability under multiple level warming treatments, we employed K‐means to compute partitional clustering in R 3.3.2 (R Foundation for Statistical Computing, Vienna, Austria, 2013). Standardized major axis tests and routines (SMATR) was used to calculate common slopes in relationships between carbon fluxes and abiotic, as well as biotic factors, under multilevel warming (SMATR 2.0; Li et al., 2017). The standardized precipitation index (SPI) was calculated by the SPI program (http://drought.unl.edu/droughtmonitoring/SPI/SPIProgram.aspx) to represent drought or moist.

## 3 RESULTS

### 3.1 Microclimate

**Figure 1:**
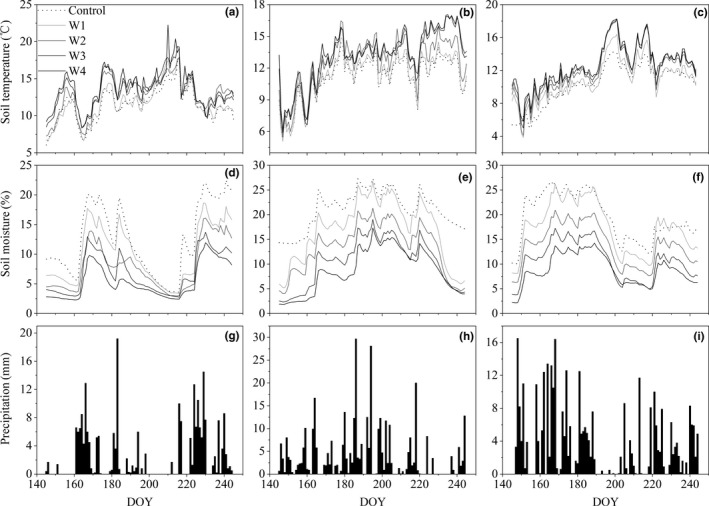
Soil temperature (°C, a–c), soil moisture (%, d–f), and precipitation (mm, g–i) during growing seasons of 2015 (left), 2016 (middle), and 2017 (right) under different warming treatments

The three growing season mean soil temperature was, on average, 0.4°C, 1.6°C, 2.0°C, 2.4°C higher in warmed plots (W1, W2, W3, W4) than control plots, respectively (Figure 1a–c). Soil moisture was lowered by 2.7%, 5.9%, 8.4%, and 10.3% under warming treatments (W1, W2, W3, W4), respectively (Figure 1d–f).

Three growing seasons were characterized by contrasting precipitation patterns. Total precipitation in 2015 (300.8 mm), 2016 (551.8 mm), and 2017 (462.2 mm) was 30.7% lower than, 27.2% higher than, and approximate to the long‐term mean annual value of 434.3 mm, respectively (Table 1; Figure 1h–i). In July of 2015, it was 63.2% (SPI = −2.2) lower than the long‐term mean (Figure 1g, Table 1). In growing season of 2016, precipitation exhibited a unimodal pattern, and July precipitation (SPI = 1.6) was 39.1% higher than the long‐term mean (Figure 1h; Table 1). In 2017, June precipitation (SPI = 1.6) was higher than the long‐term mean, while July precipitation (SPI = −1.4) was 47.5% lower than the long‐term mean (Figure 1i, Table 1). Furthermore, precipitation indices of *D*_size_ and AVG_size_ displayed more concentrated and strengthened precipitation patterns in 2016 and 2017 than in 2015 (Table 1). Compared with 2015, AVG_Int_ showed that precipitation events were less long precipitation intervals in 2016 and 2017 (Table 1).

**Table 1:** The precipitation amount and precipitation indices in growing seasons of 2015–2017

	2015	2016	2017	Mean
Precipitation amount				
April (mm)	17.5	4.1	7.8	10.0
May (mm)	24.5	37.4	69.4	32.9
June (mm)	87.5	136.8	140.3	84.4
July (mm)	38.3	144.8	54.7	104.1
August (mm)	88.5	79.7	93.6	97.0
September (mm)	27.4	100.8	90.9	69.7
Precipitation indices				
*D*_size_	5.6	8.3	7.5	–
AVG_size_	5.0	5.6	5.5	–
AVG_Int_	0.73	0.65	0.68	
1.0–5.0 mm	31.0	52.0	45.0	–
5.1–10.0 mm	19.0	19.0	23.0	–
10.1–20.0 mm	5.0	10	12.0	–
>20.1 mm	0.0	2.0	1.0	–

Note

*D*_size_: the seasonality of the precipitation event size. AVG_size_: the mean precipitation event size (Guo et al., 2015). AVG_Int_: the ratio of total length days (<1 mm) to the number of precipitation intervals. The mean was calculated from 1955 to 2017 data in the table.

### 3.2 Responses of carbon fluxes and plant coverage to the variability of precipitation under multilevel temperature increments

**Figure 2:**
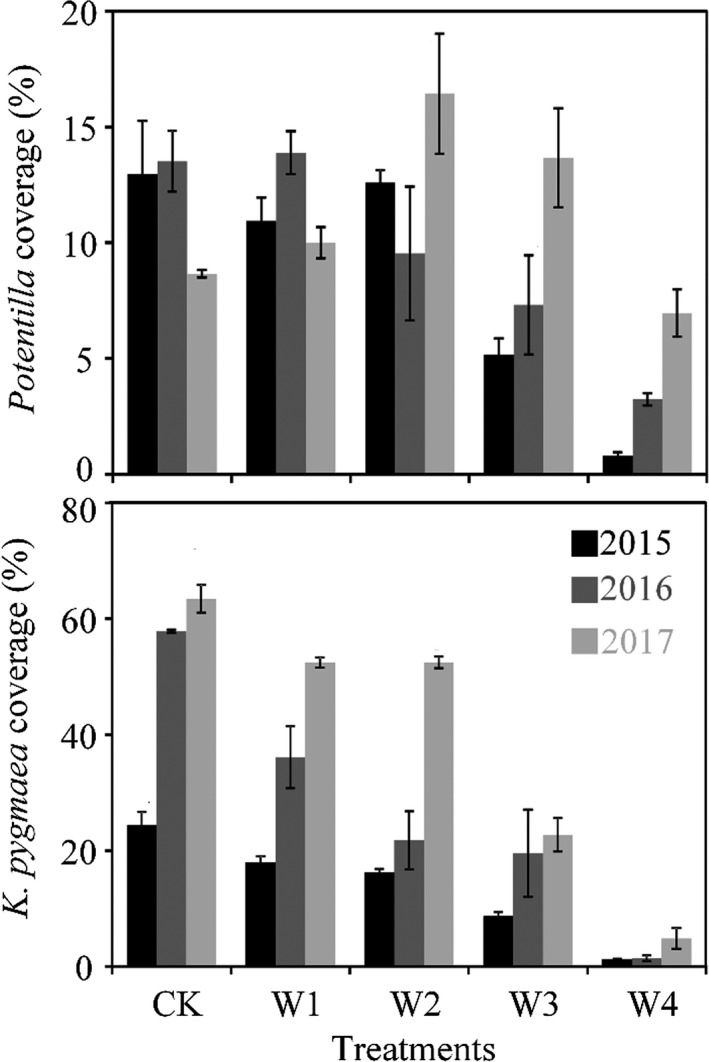
Seasonal coverage means of *Kobresia pygmaea* and *Potentilla* in the three growing seasons. CK: control

The *K. pygmaea* coverage was significantly changed during the growing seasons of 2015–2017 under control (*p* = 0.001), W1 (*p* = 0.010), and W2 (*p* = 0.000), but not W3 (*p* = 0.211) and W4 (*p* = 0.381; Figure 2; Table 2). In contrast, the significant changes in *Potentilla* coverage were found under W1 (*p* = 0.022) and W4 (*p* = 0.040), but not control (*p* = 0.295), W2 (*p* = 0.405) and W3 (*p* = 0.273; Figure 2; Table 2).

**Table 2:** Repeated measurement ANOVA analysis on the effects of time‐of‐season on *Kobresia pygmaea* and *Potentilla* coverage

	*Kobresia pygmaea*			*Potentilla*		
	*P*_Sphericity	*df*	*P_*Season	*P*_Sphericity	*df*	*P_*Season
Control	0.257	2	0.001	0.579	2	0.295
W1	0.357	2	0.010	0.761	2	0.022
W2	0.346	2	0.000	0.939	2	0.405
W3	0.018	1	0.211	0.406	2	0.273
W4	0.182	2	0.381	0.209	2	0.040

Abbreviations: *df*, degree of freedom; *P_*Season, the *p* value of tests of within‐subjects effects; *P*_Sphericity, the *p* values of Mauchly's test of sphericity.

**Figure 3:**
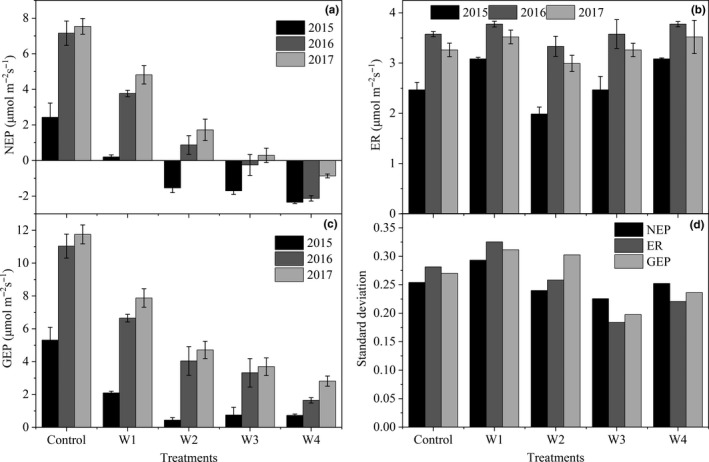
Seasonal means of NEP (a), ER (b), and GEP (c) in 2015–2017 and their standard deviation (d) among three growing seasons

Net ecosystem productivity significantly changed during the growing seasons of 2015–2017 under control (*p* = 0.003), W1 (*p* = 0.009), W3 (*p* = 0.039), and W4 (*p* = 0.003), but not W2 (*p* = 0.055; Figure 3a; Table 3). The significant variabilities in ER were found under control (*p* = 0.043), W1 (*p* = 0.005), and W3 (*p* = 0.014), but not W2 (*p* = 0.053) and W4 (*p* = 0.370; Figure 3b; Table 3). Gross ecosystem productivity exhibited significant changes under control (*p* = 0.003), W1 (*p* = 0.004), W2 (*p* = 0.040), W3 (*p* = 0.015), and W4 (*p* = 0.027; Figure 3c; Table 3).

**Table 3:** Repeated measurement ANOVA analysis on the effects of time‐of‐season on NEP, ER, and GEP

	NEP			ER			GEP		
	*P*_Sphericity	*df*	*P_*Season	*P*_Sphericity	*df*	*P_*Season	*P*_Sphericity	*df*	*P_*Season
Control	0.687	2	0.003	0.210	2	0.043	0.481	2	0.003
W1	0.352	2	0.009	0.589	2	0.005	0.109	2	0.004
W2	0.683	2	0.055	0.278	2	0.053	0.894	2	0.040
W3	0.640	2	0.039	0.388	2	0.014	0.657	2	0.015
W4	0.307	2	0.003	0.116	2	0.370	0.371	2	0.027

Abbreviations: *df*, degree of freedom; *P_*Season, the *p* value of tests of within‐subjects effects; *P*_Sphericity, the *p* value of Mauchly's test of sphericity.

**Figure 4:**
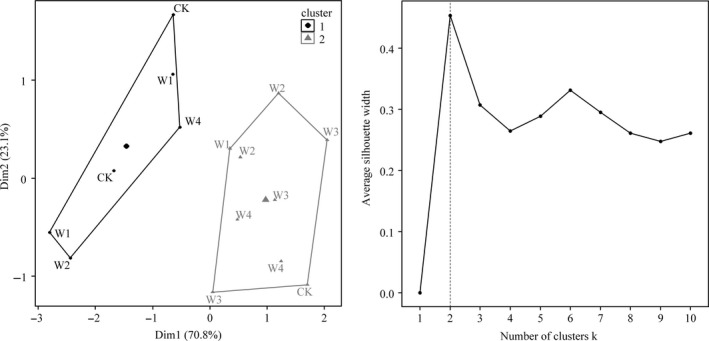
K‐means clustering analysis on the *SD* of NEP, ER, and GEP among three growing seasons (left) and the optimal number of clusters (right)

Based on the min‐max normalization, we calculated the standard deviations (*SDs*) of carbon fluxes among the three growing seasons under each warming treatment. The average *SD* of NEP, ER, and GEP under low‐level warming (control, W1 and W2) was 0.09 μmol m^−2^ s^−1^, 0.02 μmol m^−2^ s^−1^, and 0.08 μmol m^−2^ s^−1^ higher than that of high‐level warming (W3 and W4; Figure 3d). In addition, K‐means clustering results showed that *SD* of carbon fluxes among the three growing seasons under the five experimental treatments can be divided into two categories, with control and W1 being assigned to one class and W2–W4 being assigned to the other class (Figure 4).

### 3.3 Responses of carbon fluxes to decreased and increased precipitation

**Figure 5:**
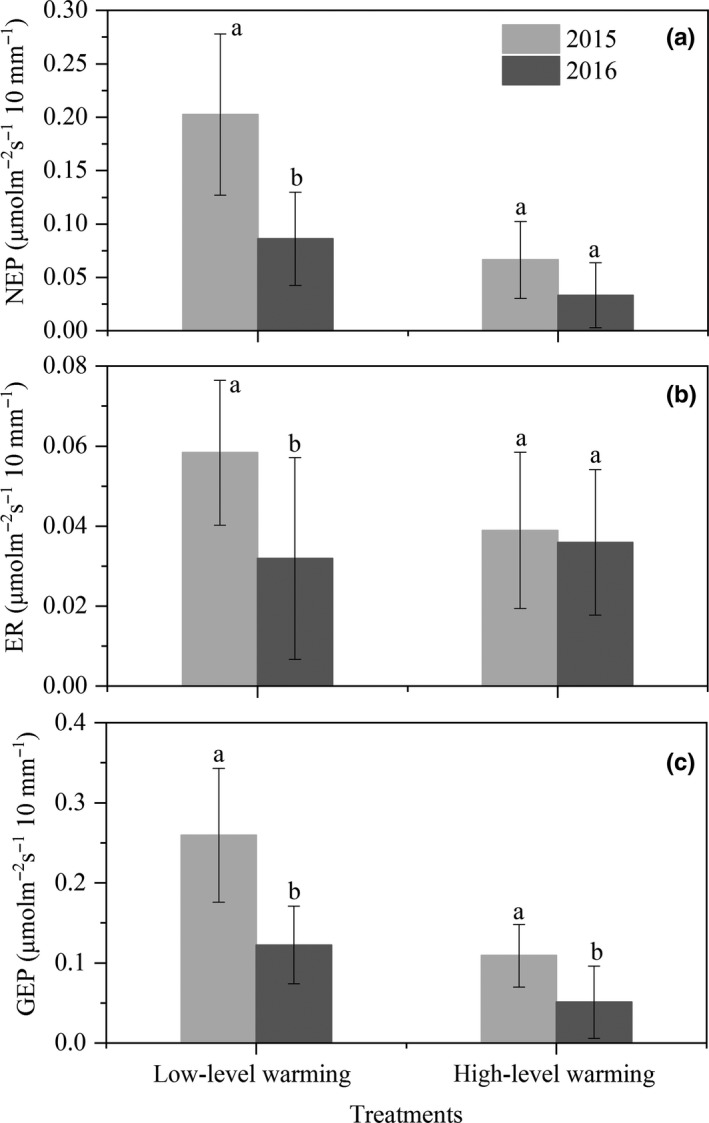
NEP, ER, and GEP sensitivity to precipitation variations (µmol m^−2^ s^−1^/10 mm) in 2015 (dry) and 2016 (wet). Different letters in insets indicate significant differences (*p* < 0.05). High‐level warming: W3 and W4; low‐level warming: control, W1, and W2

**Figure 6:**
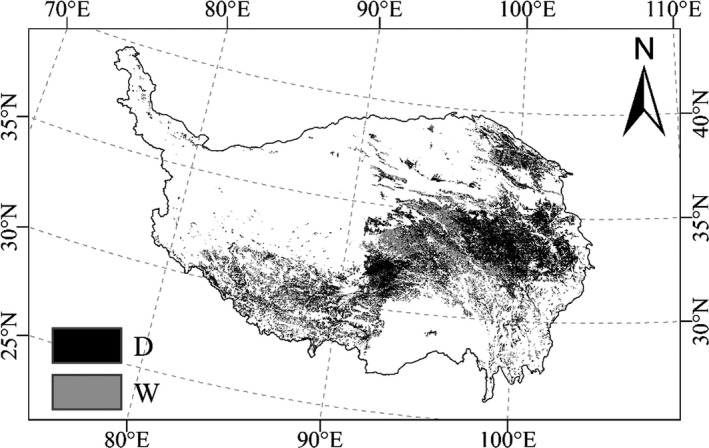
NPP sensitivity to precipitation variations (µmol m^−2^ s^−1^/10 mm) across the alpine meadow. W (gray region): positive asymmetry in NPP responses to precipitation; D (black region): negative asymmetry in NPP responses to precipitation

Based on above seasonal variability of carbon fluxes and plant coverage, five warming treatments can be divided into low‐level warming (control, W1 and W2) and high‐level warming (W3 and W4). Precipitation sensitivity of NEP, ER, and GEP in dry growing seasons was 135.1% (*p* = 0.002), 82.8% (*p* = 0.028), and 112.1% (*p* = 0.001) greater than in wet growing seasons under the low‐level warming (Figure 5; Table 4). While there was marginal difference in precipitation sensitivity of NEP (*p* = 0.149) and ER (*p* = 0.809) between dry and wet growing seasons under the high‐level warming, but not GEP (*p* = 0.041; Figure 5; Table 4), the sensitivity of carbon fluxes to dry or wet in low‐level warming was higher than that of high‐level warming (Figure 5). At the regional scale, ecosystem NPP exhibited higher sensitivity to decreased precipitation for 53.5% of alpine meadow region (Figure 6). Both the in situ measurements and ecosystem modeling results at the regional scale pointed to the negative and asymmetric responses of carbon fluxes to precipitation variations.

**Table 4:** The hypothetical test of normality and homogeneity of variances in one‐way ANOVA

Parameters		NEP		ER		GEP	
		Levene statistic	Shapiro–Wilk	Levene statistic	Shapiro–Wilk	Levene statistic	Shapiro–Wilk
Low‐level	Dry	0.135	0.965	0.506	0.492	0.072	0.691
	Wet		0.425		0.054		0.202
High‐level	Dry	0.841	0.271	0.995	0.244	0.954	0.422
	Wet		0.198		0.550		0.002

### 3.4 Impacts of biotic and abiotic factors on carbon fluxes

The seasonal variabilities of GEP and ER were mainly regulated by soil moisture and *K. pygmaea* coverage under control, W1, and W2 (Table 5; *p* < 0.05). In contrast, soil temperature and *Potentilla* coverage mostly had insignificant effects on GEP and ER under W3 and W4 (Table 5; *p* > 0.05). We further found that the slopes between biotic, abiotic factors, and GEP were steeper than that of ER in all the warming treatments (Table 5), which indicates the stronger responses of GEP to biotic and abiotic factors than that of ER.

**Table 5:** Parameters for standardized major axis estimation regressions between seasonal variability of carbon fluxes and biotic and abiotic factors in different warming treatments in 2015–2017

	GEP			ER			NEP		
	*R* ^2^	*p*	Slope	*R* ^2^	*p*	Slope	*R* ^2^	*p*	Slope
The coverage of *Potentilla*									
Control	0.00	0.90	0.77	0.17	0.23	−0.015	0.03	0.68	0.65
W1	0.00	0.70	0.96	0.03	0.67	0.20	0.02	0.71	0.78
W2	0.34	0.10	0.38	0.01	0.80	0.11	0.38	0.08	0.29
W3	0.18	0.24	0.36	0.10	0.41	0.13	0.23	0.19	0.24
W4	0.80	0.00	0.61	0.19	0.24	0.34	0.69	0.01	0.44
The coverage of *Kobresia pygmaea*									
Control	0.69	0.01	0.19	0.79	0.00	0.04	0.62	0.01	0.16
W1	0.83	0.00	0.17	0.82	0.00	0.04	0.80	0.00	0.14
W2	0.45	0.05	0.31	0.09	0.43	0.09	0.51	0.03	0.24
W3	0.22	0.20	0.27	0.24	0.18	0.10	0.19	0.24	0.18
W4	0.42	0.06	0.63	0.23	0.19	0.35	0.23	0.20	0.45
Soil moisture									
Control	0.73	0.00	0.84	0.74	0.00	0.16	0.67	0.01	0.71
W1	0.87	0.00	0.79	0.85	0.00	0.17	0.84	0.00	0.64
W2	0.62	0.01	0.88	0.60	0.01	0.26	0.55	0.02	0.68
W3	0.41	0.07	1.17	0.34	0.10	0.43	0.38	0.08	0.76
W4	0.53	0.03	0.62	0.23	0.20	0.34	0.35	0.10	0.44
Soil temperature									
Control	0.32	0.12	−7.30	0.23	0.19	−1.38	0.31	0.12	−6.16
W1	0.37	0.08	−6.96	0.41	0.06	−1.47	0.35	0.10	−5.62
W2	0.24	0.18	−6.23	0.50	0.03	−1.85	0.16	0.28	−4.78
W3	0.21	0.22	−5.07	0.34	0.10	−1.85	0.15	0.31	−3.32
W4	0.11	0.39	−2.28	0.25	0.17	−1.27	0.01	0.86	−1.63

## 4 DISCUSSION

For alpine grasslands, carbon fluxes responses to precipitation variability differed with warming magnitude. At a situ scale, carbon fluxes were much more sensitive to precipitation variability in low‐level warming than that of high‐level warming. The contrasting response pattern can be attributable to soil water availability, as well as biotic features of each species.

### 4.1 Impacts of biotic and abiotic factors on ecosystem carbon fluxes

#### 4.1.1 Impacts of precipitation variability on carbon fluxes

Precipitation is a key factor modulating ecosystem carbon processes (Biederman et al., 2016; Dong et al., 2011). Strengthened precipitation results in increased photosynthesis and transpiration rate, contributing to elevated GEP (Jia, Zha, Gong, Wang, et al., 2016; Jia, Zha, Gong, Wu, et al., 2016). Enhanced plant activity would stimulate belowground carbon input, root and microbial activities and respiration, leading to reinforced whole‐ecosystem respiration (Niu et al., 2008). Nevertheless, precipitation may impact GEP and ER differently, thus modulating NEP (Chen, Luo, Xia, Shi, et al., 2016; Chen, Luo, Xia, Wilcox, et al., 2016; Li et al., 2017). In this study, the variabilities of GEP and ER were positively related to precipitation. Consistent with previous study (Aires, Pio, & Pereira, 2008), GEP is more sensitive than ER to precipitation variability, causing increased NEP in wet growing seasons relative to dry growing season. Furthermore, temperature could regulate ecosystem responses to precipitation variability. The seasonal variability of soil moisture in the low‐level warming treatments was stronger (*CV* = 22.4%) than that in high‐level warming treatments (*CV* = 19.9%). This study revealed that soil moisture significantly affects carbon fluxes under low‐level warming treatments, but not under high‐level warming treatments. The contrasting pattern validates the interacted effects of warming and precipitation on carbon fluxes.

#### 4.1.2 Impacts of community structure on carbon fluxes

Climate change can also exert effects on ecosystem processes indirectly through climate‐mediated changes in plant species composition (Poulter et al., 2014), thereby ecosystem functions (Kulmatiski & Beard, 2013; Sala, Gherardi, Reichmann, Jobbagy, & Peters, 2012). Conventionally, shallow‐rooted plants mostly utilize shallow soil water and being highly sensitive to precipitation variations (Liu et al., 2012). Modified plant community cover would cause a series of changes in soil evaporations, autotrophic respiration, and canopy photosynthesis (Liu, Cieraad, Li, & Ma, 2016; Verburg et al., 2004). Ecosystem GEP is mainly controlled by its photosynthesis capability (Xia et al., 2015). For the alpine ecosystem, ER variations are dominated by those of autotrophic plant respiration (Chen, Luo, Xia, Shi, et al., 2016; Chen, Luo, Xia, Wilcox, et al., 2016). Considering all these interactions, precipitation plays a key role in regulating carbon fluxes.

For the alpine meadow ecosystem, *K. pygmaea,* as a dominant species, is shallow‐rooted species (Dorji et al., 2013) and relies strongly upon soil surface water (Liu et al., 2012). The coverage of *K. pygmaea* in wet growing seasons was higher than that in dry growing season under low‐level warming treatments, whereas they exhibited no significant differences in high‐level warming treatments. In parallel, carbon fluxes showed positive relationships with *K. pygmaea* coverage under low‐level warming treatments, but this relationship became nonobvious under high‐level warming treatments. This result further corroborates ecosystem structure (species composition) determined ecosystem processes (carbon fluxes).

Discriminant responses of plant species to warming also led to shifts in their relative dominance (Post & Pedersen, 2008). Under warming, coverage of dominant species (*K. pygmaea*) dramatically declined, while that of subdominant species (*Potentilla*) slightly declined. For the alpine meadow under low‐level warming treatments, coverage of *K. pygmaea* and *Potentilla* accounts for 65.5% and 23.0% of the total, respectively. Under high‐level warming treatments, these values changed to 40.7% and 42.3%, respectively. The species composition change caused corresponding carbon fluxes shifts. Compared with shallow‐rooted plants, deep‐rooted species can tap relatively deeper soil water and exhibit stronger resistance to precipitation variations (Xu & Li, 2006; Xu, Li, Xu, & Zou, 2007). For the alpine meadow, *Potentilla* is a relatively deep‐rooted plant, and its coverage exhibited marginal differences among hydrologically contrasting growing seasons. So their contribution to precipitation‐driven variability in carbon fluxes was weaker relative to the shallow deep‐rooted species (Liu et al., 2016).

### 4.2 Asymmetric responses of carbon fluxes to decreased and increased precipitation

This study revealed that NEP and its two components were much more sensitive to decreased than to increased precipitation under low‐level warming treatments, but exhibited marginal differences in high‐level warming treatments. Extreme climates cause differential survivorship among species and modify community structure and species distributions (Engelbrecht et al., 2007; Miriti, Rodriguez‐Buritica, Wright, & Howe, 2007). Drought restricts leaf emergence and canopy development, leading to decreased plant cover and increased plant mortality (Dong et al., 2011). In addition, dry air and/or soil conditions downgrade leaf stomatal conductance (Jia, Zha, Gong, Wang, et al., 2016; Jia, Zha, Gong, Wu, et al., 2016). Declined plant cover related to stomatal closure could suppress canopy photosynthetic capacity (Chen, Luo, Xia, Shi, et al., 2016; Chen, Luo, Xia, Wilcox, et al., 2016) and further restrain GEP. Suppressed root and microbial activities under drought conditions, together with reduced plant cover, lead to lowered ER (Liu et al., 2016; Niu et al., 2008).

**Figure 7:**
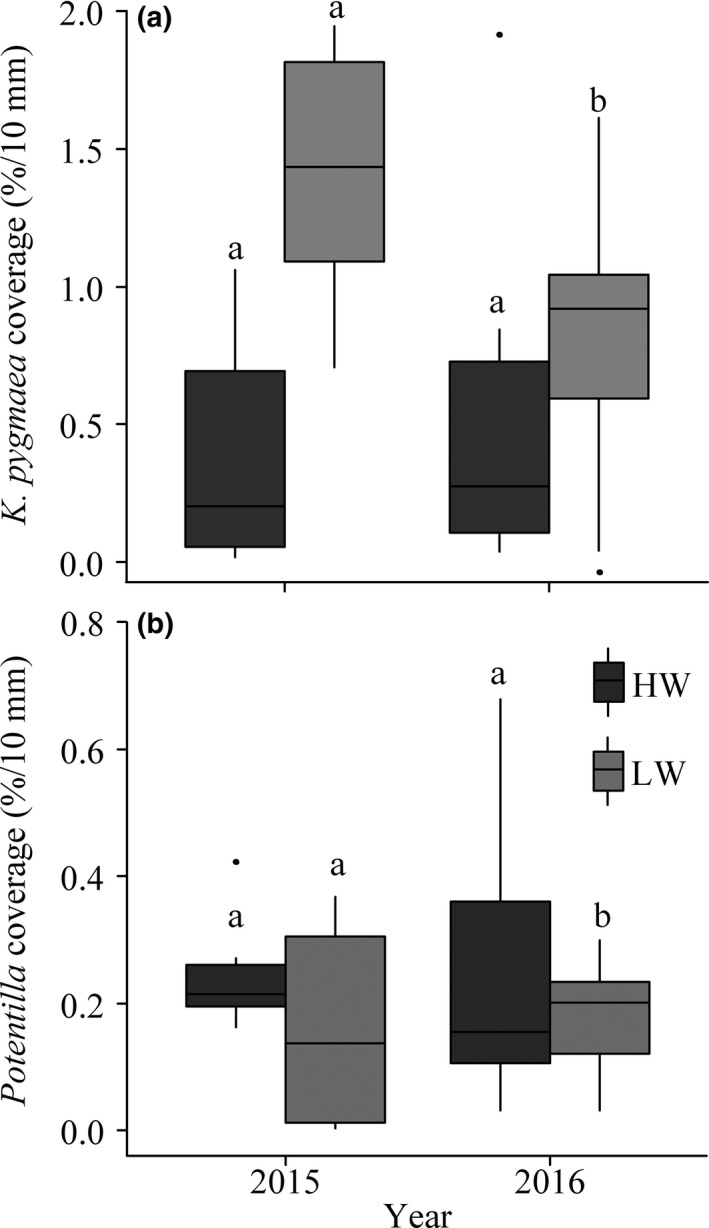
The sensitivity of *Kobresia pygmaea* and *Potentilla* coverage to precipitation variations (%/10 mm). Different letters in insets indicate significant difference (*p* < 0.05). HTI: high‐level warming treatments (W3 and W4); LTI: low‐level warming treatments (control, W1, and W2)

Drought effects vary with drought intensity, stress duration, and plant function type (McDowell et al., 2008; Welp, Randerson, & Liu, 2007). The growing season of 2015 was the driest in the 3 years. The extreme occurred between July 4 and August 10 (DOY: 185‐222), with only 34.8 mm precipitation. More importantly, *K. pygmaea* is a drought vulnerable species (Li et al., 2011), and warming further exacerbates its vulnerability. Compared with 2016 and 2017, coverage of *K. pygmaea* remarkably decreased in 2015 under low‐level warming treatments (Figure 7). Coverage of *K. pygmaea* was much more sensitive to decreased than to increased precipitation in low‐level warming (*p* = 0.001), but not high‐level warming (*p* = 0.530; Figure 7; Table 6). In contrast, *Potentilla* possesses stronger drought resistance under low‐level warming (*p* = 0.546) and high‐level warming (*p* = 0.394; Figure 7; Table 6), especially for *Potentilla bifurca Linn* (Wei & Li, 2003). The positively linear correlation between *K. pygmaea* coverage and carbon fluxes, together with the asymmetrical response of *K. pygmaea* to hydrologically contrasting conditions, resulted in negative asymmetry in carbon fluxes responses to precipitation in low‐level warming treatments.

**Table 6:** The hypothetical test of normality and homogeneity of variances in one‐way ANOVA

Parameters		*Kobresia pygmaea*		*Potentilla*	
		Levene statistic	Shapiro–Wilk	Levene statistic	Shapiro–Wilk
Low‐level	Dry	0.246	0.056	0.709	0.152
	Wet		0.242		0.003
High‐level	Dry	0.426	0.674	0.043	0.114
	Wet		0.639		0.203

Climate influences ecosystem productivity by adjusting vegetation phenology (Pau et al., 2011). Although plentiful precipitation fell in August of a dry growing season in 2015, its effects on carbon fluxes are marginal (Craine et al., 2012). The ecosystem productivity variation in August is highly related to late July precipitation (Craine et al., 2012). In the late growing season, solar radiation weakens and photosynthetic capacity of old leaves subsides (Gunderson et al., 2012). An advanced leaf emergence in spring exerts greater influences on seasonal carbon uptake than an equivalent delay duration of fall senescence (Marchin, Salk, Hoffmann, & Dunn, 2015). This phenomenon can also be explained by the “slow in, rapid out” principle of net ecosystem exchange. The negative anomalies in precipitation may induce drought‐stress mortality. Recovery from the drought through regeneration may be slow, even under adequate precipitation (Korner, 2003). Therefore, negative asymmetry response is also explained by the seasonal distribution of precipitation in a dry growing season.

## 5 CONCLUSIONS

Carbon fluxes in low‐level warming treatments were more sensitive to precipitation variability than that in high‐level warming treatments for the alpine meadow ecosystem. Such distinctions were ascribable to different in soil water availability and dominances of shallow‐rooted plant under low‐ and high‐level warming treatments. Furthermore, carbon fluxes respond more strongly to decreased than to increased precipitation, leading to their negative and asymmetric responses. This study highlights that the interannual variations of precipitation play a critical role in modulating ecosystem carbon cycling, whereas this effect varies with warming magnitude.

## CONFLICT OF INTEREST

None declared.

## AUTHOR CONTRIBUTIONS

Ning Chen and Yangjian Zhang conceived and designed the study. Ning Chen, Junxiang Li, Yaojie Liu, Juntao Zhu, Ze Tang, Li Wang, and Nan Cong contributed field measurements. Ke Huang and Jiaxing Zu contributed the space validation. Ning Chen and Yangjian Zhang wrote the paper. All authors contributed to the writing the manuscript. The authors declare no conflict of interest.

## Data Availability

I agree to deposit the main data of the article in Dryad. https://doi.org/10.5061/dryad.506p9k0.
